# First Draft Genome Sequence of *Balamuthia mandrillaris*, the Causative Agent of Amoebic Encephalitis

**DOI:** 10.1128/genomeA.01013-15

**Published:** 2015-09-24

**Authors:** H. Detering, T. Aebischer, P. W. Dabrowski, A. Radonić, A. Nitsche, B. Y. Renard, A. F. Kiderlen

**Affiliations:** a Research Group Bioinformatics (NG4), Robert Koch Institute, Berlin, Germany; b Division for Mycotic, Parasitic and Mycobacterial Diseases (FG16), Robert Koch Institute, Berlin, Germany; c Centre for Biological Threats and Special Pathogens (ZBS1), Robert Koch Institute, Berlin, Germany

## Abstract

The free-living amoeba *Balamuthia mandrillaris* is a rare but highly lethal agent of amoebic encephalitis in humans and many other mammalian species. Here, we announce the first draft genome sequence of the original 1990 isolate cultured from the brain of a deceased mandrill baboon.

## GENOME ANNOUNCEMENT

*Balamuthia mandrillaris* is an opportunistic parasite that causes amoebic encephalitis (BAE) in humans and other mammals (1, 2), with a fatal outcome rate of >95%. The amoeba appears to be ubiquitous in soil (3–5) and freshwater habitats (6), and it invades its hosts via pulmonary (7), nasal/olfactory nerve (8), cutaneous (9), and possibly gastrointestinal (10) routes. BAE has been reported in immunocompromised (11) patients and in children and adults without clinical evidence for acquired or innate immunodeficiency (12, 13). Experimental infections in mice lacking lymphocyte subsets show that CD4^+^ T cells are important for resistance to intranasal *B. mandrillaris* infection and BAE (14).

Axenic cultivation of the CDC-V039 isolate was performed in modified Chang’s special medium at 37°C in a humidified normal atmosphere supplemented with 5% CO_2_ (15). Genomic DNA was isolated with the Qiagen DNeasy blood and tissue kit, according to the manufacturer’s instructions. Genome sequencing was performed at the Robert Koch Institute on Roche 454 FLX, Illumina HiScan, and Illumina HiSeq 1500 machines and on a Pacific Biosciences *RS* II at the University of Lausanne (UNIL), Switzerland, with 20×, 1,600×, and 90× read coverage, respectively. Genome assembly of 756,805 PacBio reads with the HGAP3 (16) pipeline, followed by quality filtering of contigs, resulted in a genome of 68 Mbp in 1,605 contigs, with an *N*_50_ value of 93,953 bp and an average G+C content of 46.8%. An integrated analysis using Illumina, 454, and PacBio data revealed a complex genome with indication of hyperploidy. A comparison of the mitochondrial genome structure and homology searches of *Acanthamoeba castellanii* protein sequences in the newly assembled genome suggest a more distant relationship of *B. mandrillaris* to its closest known relative than was previously assumed. Functional genome annotation is in progress and will help unravel the metabolic and pathogenic potential of this opportunistic parasite.

### Nucleotide sequence accession numbers.

The draft genome sequence of *B. mandrillaris* CDC-V039 has been deposited in GenBank under the accession no. LFUI00000000. The version described in this paper is the first version, LFUI01000000.
